# Special Issue: Molecular Research on Dental Materials and Biomaterials 2018

**DOI:** 10.3390/ijms21239154

**Published:** 2020-12-01

**Authors:** Ihtesham Ur Rehman, Mary Anne Melo

**Affiliations:** 1 Engineering Department, Faculty of Science and Technology, Lancaster University, Gillow Avenue, Lancaster LA1 4YW, UK; i.u.rehman@lancaster.ac.uk; 2 Division of Operative Dentistry, Department of General Dentistry, University of Maryland School of Dentistry, 650 W. Baltimore St, Baltimore, MD 21201, USA

Worldwide, populations of all ages suffer from oral diseases, disorders, pathological conditions of the oral cavity, and their impact on the human body. New dental materials and biomaterials, currently being introduced, under development, or envisioned, are expected to benefit the oral health status. These materials will provide a wide range of diverse functions, from promoting osteogenesis to bacterial biofilm formation inhibition.

Factually, dental biomaterials were intended to provide core functions, such as mechanical support to masticatory loads (e.g., dental crowns) or optical properties to display a pleasant and natural appearance (e.g., resin composites). This approach has led to the successful design of numerous clinically used materials over the years, such as sealants, orthodontic adhesives, luting cement, hybrids materials for computer-assisted design/computer-assisted manufacturing (CAD/CAM)-based restorative dentistry.

However, despite the ongoing development, unmet dental needs remain. The mouth is a dynamic environment, so dental materials regularly experience changes that alter performance, and this highlights the profound need for methods to allow tracking of its performance in physiologically complex especially simulating the intraoral environment. Both novel and cutting-edge preventive and therapeutic strategies are still in a claim.

In the special issue “Dental Materials and Biomaterials 2018”, encouraging findings on progressing treatment of the most prevalent oral conditions were described, covering different aspects of technological development to treatment options, considering insights derived from a plethora of in vitro/ in situ models and clinical trials.

In efforts toward caries prevention, Ibrahim et al. [1], Zhou et al. [2], and Hao et al. [3] explored the application of dimethylaminohexadecyl methacrylate (DMAHDM) as an antibacterial strategy for resin-based materials. Barszczewska-Rybarek et al [4] also have explored the silver ions releasing approach intended for dental materials. Bioactive dimethacrylate composites filled with silver nanoparticles (AgNP) might be used in medical applications, such as dental restorations and bone types of cement. DMAHDM, an antibacterial monomer, acts primarily through direct contact killing. It has been previously hypothesized that a positively charged DMAHDM structure may interact with negatively charged bacteria to lead to cell membrane leakiness and rupture [1]. Various innovative methods have been applied to develop dental adhesives with particular functions to tackle these problems, such as incorporating matrix metalloproteinase inhibitors, antibacterial or remineralizing agents [3] into bonding systems, as well as improving the mechanical/chemical properties of adhesives, even combining these methods.

The targeted cariogenic biofilm is a densely packed community of oral microbial cells with predominantly acid-tolerant, acid-producing bacteria surrounded by an exopolysaccharide (EPS)-rich matrix. Within cariogenic dental plaque, Streptococcus mutans (S. mutans) has been strongly linked to cariogenic biofilm formation and carious lesion progression.

Delgado-Ruiz and Romanos [5] reviewed the current knowledge on titanium particles and ions released from metallic instruments used for implant bed preparation, from the implant surfaces during insertion implant-abutment interface during insertion and functional loading. Besides, the implant surfaces and restorations are exposed to the environment, saliva, bacteria, and chemicals that can potentially dissolve the titanium oxide layer. If these agents attack continuously, the implant surface can permanently lose its titanium oxide layer.

The formation of soluble compounds on the titanium surface will alter the implant surface chemistry and facilitate the dissolution and degradation of exposed bulk titanium, resulting in corrosion cycles. Implant maintenance procedures can potentially alter implant surfaces and produce titanium debris released into the peri-implant tissues. While this treatment is one of the most popular, side effects constrain its use.

Xie et al [6] have shown that graphene promotes osteogenesis via the activation of the mechanosensitive integrin/FAK axis future therapy to overcome this problem. Another possibility of improved osseointegration is discussed and reviewed by Marco Lupi et al. [7]. Biochemical modification of titanium surfaces (BMTiS) entails the immobilization of biomolecules to implant surfaces to induce specific host responses. This crossover randomized clinical trial assesses the clinical success and marginal bone resorption of dental implants bearing a surface molecular layer of covalently linked hyaluronan compared to control implants up to 36 months after loading.

In this special issue, insightful progress for dental material and biomaterials were described, and new avenues for application and translation were discussed. Particular emphasis was placed on dental materials—structure-property correlations in the context of both the clinical and non-clinical aspects. These papers provide readers with the necessary tools and principles of Dental Materials and biomaterials currently used in Clinical Dentistry and cover the underlying principles of bioactivity and biocompatibility.

Furthermore, Karakida et al. [8] studied drug repositioning is the process of discovering, validating, and marketing previously approved drugs for new indications. Due to the promise of reduced costs and expedited approval schedules, the research field of drug repositioning has been attracting attention. A standard database consisting of both approved and failed drugs has been developed as a web application to find candidates for drug repositioning. Without using the database, the present study demonstrated that MDZ enhances the differentiation of PPU-7 cells to odontoblast and promotes dentin-like hydroxyapatite formation. Further studies are required to elucidate the pharmacokinetics and pharmacological efficacy of MDZ in animal experiments. In the dental field, these findings support the repositioning of MDZ to promote dentin regeneration for endodontic treatments, such as pulp capping. Moreover, these findings support advancing research from pig to human experimental models using human DPSCs to discover MDZ’s potential, not only for future dental treatments, but also for regenerative organ medicine. This special issue offers an exciting background of MS pathophysiology and disease development and prediction from animal models and clinical studies and suggests strategies for developing MS therapies.
